# Heterogeneity of Active Sites in the Polymer Chain Transfer Reactions at Olefin Polymerization over Multisite Supported Ziegler-Natta Catalysts

**DOI:** 10.3390/polym15214316

**Published:** 2023-11-03

**Authors:** Mikhail Matsko, Vladimir Zakharov

**Affiliations:** Boreskov Institute of Catalysis, Pr. Lavrentieva 5, Novosibirsk 630090, Russia

**Keywords:** olefin polymerization, chain transfer reaction, polyethylene, polypropylene, polyolefins, polyhexene-1, active sites, molecular weight, molecular weight distribution titanium–magnesium catalysts, copolymerization

## Abstract

In this review, we summarize and discuss our experimental data published in a number of papers on the transfer reactions of polymer chains in the polymerization of ethylene, propylene, and hexene-1, and the copolymerization of ethylene with α-olefins over multisite supported titanium–magnesium catalysts (TMC). Three groups of transfer reactions are discussed in the review: (1) transfer reactions with AlEt_3_ cocatalyst, (2) transfer reactions with hydrogen, and (3) transfer reactions with participation of α-olefins in the case of ethylene copolymerization with α-olefins. We have found polymerization conditions where it is possible to observe heterogeneity of active sites of TMC for all three groups of the indicated reactions. It is shown that (1) the transfer reaction with AlEt_3_ proceeds with higher reactivity on the active sites that produce polymers with low molecular weight; (2) the transfer reaction with hydrogen, in the case of α-olefin polymerization and copolymerization of ethylene with α-olefins, proceeds with higher reactivity on the active sites which produce polymers with high molecular weight; (3) the transfer reaction with α-olefins proceed with higher reactivity on the active sites that produce high molecular weight polymers.

## 1. Introduction

Transfer reactions of a growing polymer chain can largely determine the molecular weight and molecular weight distribution of the polymers formed during polymerization. Polymers with broad molecular weight distribution (MWD) and different polydispersity values (M_w_/M_n_ = 3–25) are formed in the case of olefin polymerization over various commercial catalysts (e.g., supported Ziegler–Natta catalysts, chromium oxide catalysts with silica support) due to heterogeneity of the active sites of these catalysts in the reactions of polymer chain propagation and transfer of polymer chain (multisite catalysts). In the case of polymerization of olefins on supported Ziegler–Natta catalysts, it is assumed that the heterogeneity of the active centers of these catalysts is associated with the heterogeneous composition and structure of the active centers and the influence of a number of additional components introduced into the composition. These active centers include a titanium compound and some additives fixed on the surface of the support (magnesium dichloride). As a result of heterogeneity of active sites TMC produce polyethylene (PE) with a broad molecular weight distribution (MWD) (M_w_/M_n_ = 5–8). Some data on the MWD of polyethylene produced over Ti-based catalysts are presented in the review [1] and in refs. [2,3,4,5,6,7,8,9,10,11,12,13,14]. Varying the composition of supported Ziegler–Natta catalysts makes it possible to control the heterogeneity of the active centers of these catalysts in the reactions of polymer chain growth and transfer and to obtain polyolefins with different polydispersity [15,16,17,18,19,20,21,22,23,24,25,26,27,28,29,30].

**Figure 1:**
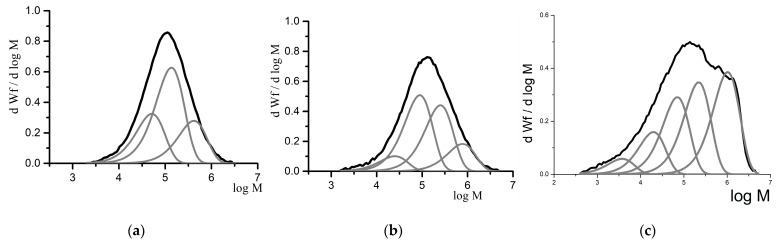
Deconvolution of MWD curves of polyethylene, produced over supported catalysts of different composition: (**a**) titanium–magnesium catalyst (0.07 wt.% of Ti, M_w_/M_n_ = 3.3), (**b**) titanium–magnesium catalyst (3 wt.% of Ti, M_w_/M_n_ = 5.0), (**c**) vanadium–magnesium catalyst (3.2 wt.% of V, M_w_/M_n_ = 16).

The analysis of the molecular weight distribution of polyolefins with a broadened MWD (polydispersity M_w_/M_n_ ≥ 3) produced over multisite catalysts is performed by the deconvolution of MWD curves into individual components with polydispersity M_w_/M_n_ = 2 (Flory components), which correspond to the polymer formed on the single site catalysts that contain only one type of active sites [4,5,6,9,10,31,32]. In this case, the number of Flory components corresponds to the number of individual types of active sites that are present in multisite catalysts and exhibit different reactivity in the polymer chain propagation and transfer reactions. By way of example, Figure 1 shows the data reported in refs. [19,33] on the deconvolution of MWD curves into Flory components for three polyethylene samples obtained over the multisite supported Ziegler-type catalysts with different compositions of the active component. It is seen that ethylene polymerization over the titanium–magnesium catalyst (TMC) with a very low titanium content results in the formation of polyethylene with quite a narrow MWD (M_w_/M_n_ = 3.3). The MWD curve for this polymer can be represented by three Flory components corresponding to three groups of active sites in this catalyst. On the TMC with an increased titanium content (3.5 wt.%), the polymer with a broader MWD (M_w_/M_n_ = 5.0) is formed. The MWD curve for this polymer can be represented by four Flory components corresponding to four groups of active sites in this catalyst. Polyethylene with the most broad and bimodal MWD is produced over the supported vanadium–magnesium catalyst (M_w_/M_n_ = 16). The MWD curve for this polymer can be represented by five Flory components corresponding to the presence of five groups of active sites in the catalyst.

According to the numerous physicochemical studies and theoretical calculations (see as example refs. [34,35,36,37]), different types of the surface titanium species are formed in titanium–magnesium catalysts firstly at the interaction of TiCl_4_ with the support—MgCl_2_, and then after interaction of these surface species with AlR_3_ cocatalysts. In particular, mononuclear Ti^3+^ species and binuclear [Ti^3+^]_2_ are formed on the different local surface centers of MgCl_2_. The addition of the stereoregulating electron donor organic compounds into the titanium–magnesium catalysts leads to the formation of a new surface species. It is known (see [38]) that only a small part of the surface titanium species (less than 10%) appears as active centers; therefore, until now, questions on the composition and structure of active sites of TMC have still been open for discussion.

At the same time, in our works [19,23,24,25,27,28,29,30,33] it was found that in some cases, when using a catalyst of the same composition, the introduction of polymer chain transfer agents into the reaction medium also leads to a change in the polydispersity of the obtained polyolefins. We believe these results are related to the additional heterogeneity of the active sites of supported Ziegler–Natta catalysts in polymer chain transfer reactions, as the composition of the reaction medium changes during polymerization.

It is necessary to note the results presented in [19,23,24,25,28,29,30,33] were obtained at different times (2009–2023) for various polyolefins (polyethylene, polypropylene, polyhexene, and ethylene-α-olefin copolymers) using supported catalysts Ziegler–Natta of various compositions. The generalization of these results, in the form of a short review, makes it possible to more reasonably formulate a new approach to control the polydispersity of polyolefins obtained over these catalysts by varying the composition of the reaction medium (polymerization conditions).

The indicated heterogeneity of active sites manifests itself upon changes in the reaction medium composition during polymerization and makes an additional contribution to the polymer polydispersity, which is determined primarily by the composition and structure of the catalyst active component and also by the structural characteristics of supported catalysts.

## 2. Materials and Methods

Data on the preparation procedures and composition of TMC used for ethylene polymerization and copolymerization of ethylene with α-olefins are presented in refs. [19,29]. Data on the preparation procedure and composition of TMC, which contains the stereoregulating electron donor compound (dibutylphthalate) used for the polymerization of propylene and hexaene-1, are presented in ref. [29]. Procedures for ethylene polymerization and copolymerization of ethylene with α-olefins are described in refs. [23,24]. Procedures for propylene and hexene-1 polymerization are presented in refs. [29]. Additional data on the polymerization conditions are reported in the corresponding Tables.

The polymer MWD determination is described in [29]. Deconvolution of MWD curves was performed according to procedures described elsewhere [19,33].

Comonomer content in copolymers of ethylene with α-olefins and content of vinyl, vinylidene, and trans vinylene groups was measured by FTIR according to [23,24].

## 3. Results and Discussion

**Scheme 1:**
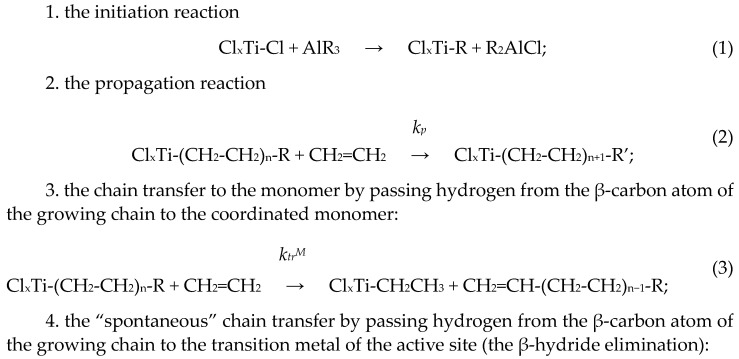

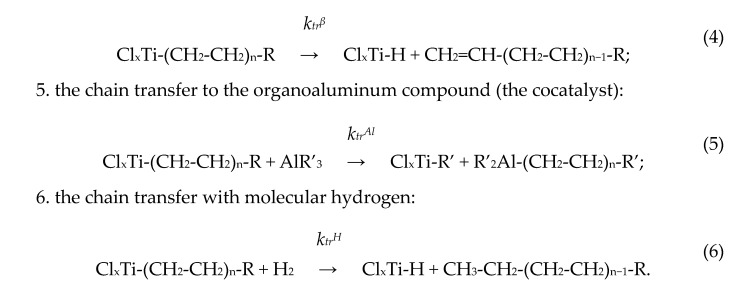
The polymer chain propagation and transfer reactions occurring during polymerization of olefins, using ethylene as an example.

The general kinetic scheme proposed by G. Natta [39] for the propylene polymerization over Ziegler–Natta catalysts (Scheme 1) is usually used for the kinetic analysis of catalytic polymerization of olefins. This scheme has been discussed in many papers [39,40,41,42,43,44,45,46,47,48,49,50,51,52,53].

Scheme 1 includes the initiation reaction, the polymer chain propagation reaction, and four polymer chain transfer reactions, which together determine the molecular weight of the produced polymers:

After chain transfer reactions, the active species Cl_x_Ti-R and Cl_x_Ti-H can react with ethylene, and the process of formation of a new polymer chain is started again (re-initiation of active sites). In some cases, polymerization can be terminated (for example, via reactions with impurities, etc.). We do not discuss the termination processes in this review.

In ref. [54] it was shown that for supported Ziegler–Natta catalysts, the *k_tr_^β^*/*k_tr_^M^* value was close to zero, and this reaction virtually did not occur under the conditions of suspension and gas-phase polymerization of ethylene.

This review summarizes data obtained in our studies [19,23,24,25,28,29,30,33] on the heterogeneity of active sites in multisite supported titanium–magnesium catalysts under the polymerization of olefins for three groups of polymer chain transfer reactions:

1. chain transfer reactions with a cocatalyst (AlEt_3_) at ethylene and hexene-1 polymerization;
2. chain transfer reaction with participation of α-olefins in the case of copolymerization of ethylene with α-olefins;
3. chain transfer reaction with hydrogen in the polymerization of ethylene, propylene, and hexene-1, and copolymerization of ethylene with α-olefins.

### 3.1. Polymer Chain Transfer Reactions Involving Aluminum Trialkyls during Polymerization of Ethylene and Hexene-1 over Supported Titanium–Magnesium Catalysts

It is generally accepted that the formation of active centers of a titanium–magnesium catalyst occurs as a result of the interaction of TiCl_4_, adsorbed on the surface of the MgCl_2_ support, with the organoaluminum cocatalyst [43,55]. The result of this interaction is the reduction of Ti^4+^ to Ti^3+^ and further alkylation of TiCl_3_, with the formation of a Ti-C bond.

Experimental data on the heterogeneity of active sites of TMC in the chain transfer reactions with triethylaluminum (TEA), during the polymerization of ethylene and hexene-1 on these catalysts, are presented in refs. [19,25,28]. These data were obtained during polymerization in the absence of hydrogen, under conditions when the transfer reaction of the polymer chain with TEA was the predominant transfer reaction.

#### 3.1.1. Chain Transfer Reactions Involving Triethylaluminum at Ethylene Polymerization

In ref. [19], it was demonstrated that in the case of polymerization of ethylene in the absence of hydrogen and at a low pressure of ethylene (lower than 4 bar), the transfer reaction of the polymer chain with TEA is the predominant transfer reaction. In these conditions, a decrease in the pressure of ethylene leads to a decrease in the molecular weight of polyethylene, as well as to an increase in the polydispersity of the resulting polymer (an increase in the M_w_/M_n_ values from 4.8 up to 9–11, experiments 1–3 in Table 1). In the case of ethylene polymerization under higher pressure of ethylene (4 bar), an increase in the concentration of TEA from 1.2 to 4.8 mmol/L also leads to a decrease in the molecular weight of polyethylene and an increase in polydispersity (an increase in M_w_/M_n_ values, experiments 4 and 5 in Table 1).

**Scheme 2:**
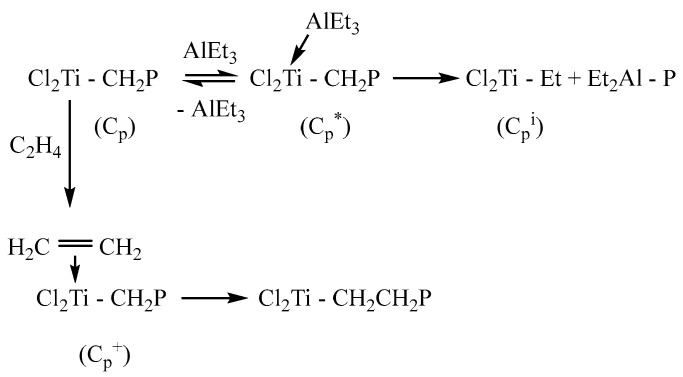
Transformations of the active sites in the polymer chain transfer reactions involving TEA during ethylene polymerization over titanium–magnesium catalysts, where C_p_ is the active site containing the coordinatively unsaturated Ti^3+^ ion bound to the growing polymer chain (–CH_2_P); C_p_^+^, the active site with coordinated ethylene; C_p_*, the temporarily inactive site containing AlEt_3_, which is adsorbed on the Ti^3+^ ion; and C_p_^i^, the active site formed via the exchange of alkyl ligands to produce the Et_2_Al–CH_2_P.

Table 2 and Table 3 show the results of the deconvolution into Flory components for the MWD curves of the polyethylene samples from the experiments listed in Table 1. It was seen that with a decrease in the ethylene pressure and increase in TEA concentration the number of Flory components increased from four components up to six components due to the appearance of new low-molecular weight components V and VI and a decrease in the contribution of components I–III. Thus, polymerization at a low ethylene pressure and high TEA concentration, when the predominant transfer reaction is the transfer with AlEt_3_, is characterized by a greater heterogeneity of active sites. We believe these results can be attributed to the formation of temporarily inactive sites containing the titanium–polymer bond during polymerization [8,12,14]. These sites emerge in the polymerization due to the reversible adsorption of TEA on the active sites containing the growing polymer chain. In this case, transformations of the active sites during polymerization and chain-growth limiting reactions (the transfer reactions involving TEA) can be represented by Scheme 2. Presumably, during polymerization, triethylaluminum competes with ethylene in the adsorption processes on the coordinatively unsaturated titanium ion in the active site (C_p_), and further transformations of the active site proceed by two routes: (1) the formation of the ethylene π-complex with the titanium ion (C_p_^+^), followed by the insertion of coordinated ethylene into the active Ti–CH_2_P bond (the chain propagation reaction); (2) the formation of the AlEt_3_ complex with the titanium ion (the temporarily inactive C_p_* site). This site can transform into the initial active site (C_p_) due to AlEt_3_ desorption, or into a new active site (C_p_^i^) as a result of chain transfer with triethylaluminum. Evidently, the concentration of the temporarily inactive sites (C_p_*) depends on the concentration of AlEt_3_ and ethylene, and will increase with an increase in the AlEt_3_ concentration and a decrease in the ethylene concentration.

We believe that the formation of the temporarily inactive C_p_* site containing the Ti–CH_2_P bond and the adsorbed TEA molecule, on which the chain propagation is interrupted, can be considered as an additional chain-growth limiting reaction (the chain transfer reaction). The data obtained show that this reaction proceeds predominantly on the active sites producing low-molecular weight polyethylene, which leads to a considerable broadening of MWD (an increase in the M_w_/M_n_ value).

In some cases, the reaction between the cocatalyst and catalyst results in deeper reduction of Ti^4+^ to Ti^2+^ which might be an additional explanation of heterogeneity of active sites [55,56].

#### 3.1.2. Chain Transfer Reactions Involving Triethylaluminum during Polymerization of Hexene-1

Polymerization of higher alpha olefins (1-hexene and 1-octene) over supported titanium–magnesium catalysts (TMC) is of significant fundamental and practical interest [57,58,59,60,61,62,63,64]. In our studies [25,65,66,67], data were obtained on the polymerization kinetics of hexene-1 over supported titanium–magnesium catalysts and the molecular weight characteristics of the produced polyhexene. It should be noted that polyhexene is an X-ray amorphous polymer soluble in heptane under polymerization conditions (solution polymerization). As was noted in our publication [30], this is one of the causes explaining essential differences in the molecular weight characteristics between polyhexene (PH) produced over TMC and polypropylene (PP) or polyethylene (PE) produced over similar catalysts by suspension polymerization as solid particles. When polyhexene is formed by solution polymerization, the TEA concentration on the catalyst surface corresponds to its concentration in the polymer solution, whereas upon formation of semicrystalline PP and PE particles by suspension polymerization, the TEA concentration on the catalyst surface may be substantially lower compared to its concentration in heptane. This may explain data presented in ref. [66], that the molecular weight of polyhexene decreases considerably with an increase in the TEA concentration during polymerization in the absence of hydrogen and at a high concentration of the monomer (2 mol/L). Thus, under the indicated conditions, TEA is an efficient and main transfer agent of the polymer chain. At the same time, polyhexene with very high molecular weight and narrow MWD (M_w_/M_n_ = 3.1) is formed at hexene-1 polymerization over TMC with tri-iso-butylaluminium (TIBA) as cocatalyst [25].

Table 4 illustrates data on the deconvolution of MWD curves into Flory components for the polyhexene samples obtained using TEA and TIBA cocatalysts. These data indicate the occurrence of three types of active sites on the TMC surface in the presence of TIBA cocatalyst. The use of the TEA cocatalyst leads to a sharp decrease (by a factor of 3–4.5) in the molecular weight of polyhexene formed on these sites and the appearance of two additional Flory components with a very low molecular weight (6 and 28 kg/mol). This may be related to the additional formation of two new groups of active sites due to the reversible adsorption of TEA on a part of active sites. This results to the temporarily interruption of the chain propagation on these sites (Scheme 2 in Section 3.1.1). This conclusion corresponds to the results presented above in Section 3.1.1 (Table 2 and Table 3), which were obtained at ethylene polymerization over supported TMC under the conditions when chain transfer in the presence of TEA was the predominant polymer chain transfer reaction.

The presented data on the polymer chain-growth limiting reactions with TEA during polymerization of ethylene (Section 3.1.1) and hexene-1 (Section 3.1.2) over supported TMC under the conditions when these reactions are the predominant chain transfer reactions suggest that the indicated reactions proceed as a complicated process shown in Scheme 2. The process includes two reactions limiting the polymer chain growth: (1) the exchange of alkyl ligands in the (C_p_*) complex in Scheme 2, which contains the active site—a titanium compound with the growing polymer chain and the TEA molecule adsorbed on the active site; (2) the interruption of the polymer chain growth due to the reversible adsorption of TEA on the active site (the C_p_* complex in Scheme 2). The desorption of TEA from the (C_p_*) complex leads to the regeneration of the active site and resumption of the polymer chain growth.

The data obtained suggests that reaction (2) occurs predominantly on the active sites producing a low-molecular weight polymer. Such heterogeneity of active sites in this reaction leads to the broadening of MWD of the formed polymers (polyethylene and polyhexene).

### 3.2. Chain Transfer Reaction Involving α-Olefins in the Case of Ethylene Copolymerization with α-Olefins over Supported Titanium–Magnesium Catalysts

Copolymerization of ethylene with α-olefins makes it possible to obtain a wide set of polymers with a decreased density compared to homopolyethylene due to the appearance of branchings in copolymer as a result of α-olefin addition to the growing polyethylene chain. At the same time, it was revealed [23,24,68] that ethylene copolymerization with α-olefins over titanium–magnesium catalysts results in the formation of the polymer with a lower molecular weight compared to homopolyethylene. The example data on the molecular weights of homopolyethylene and copolymers of ethylene with propylene and hexene-1 are presented in Table 5. So, it is necessary to propose the additional chain transfer reaction with the participation of α-olefins proceeds at copolymerization of α-olefins. The possible routes of this reaction are discussed in ref. [24] on the base of experimental data on the content of terminal double bonds of different types formed in the homopolyethylene and copolymers of ethylene with α-olefins (Table 5).

In all cases, the produced polymers contain terminal vinyl groups in the amount from 0.14 to 0.24 groups per one polymer chain, which indicates the occurrence of the chain transfer with ethylene under the given conditions (reaction (3) in Scheme 1). At the same time, ethylene copolymerization with propylene is accompanied by an increase in the content of terminal vinylidene groups and the appearance of trans-vinylene groups in ethylene copolymers with propylene and hexene-1. The formation of terminal vinylidene groups is associated with the β-hydride transfer from the β-carbon atom (*C) in the alkyl fragment obtained after 1,2-addition of propylene or hexene to the growing polyethylene chain (reaction (7)):
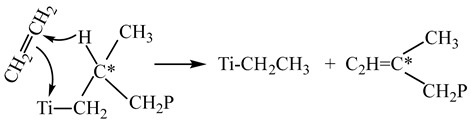
(7)

The formation of trans-vinylene groups may proceed during the β-hydride transfer from the β-carbon atom (*C) in the alkyl fragment obtained after the 2,1-addition of propylene or hexene-1 to the growing polyethylene chain (reaction (8)):
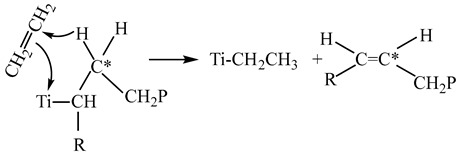
R=CH_3_, C_4_H_9_                         (8)

Thus, the decrease in the molecular weight of copolymers produced by copolymerization of ethylene with α-olefins over TMC is caused by the appearance of two new additional routes of the β-hydride chain transfer by reactions (7) and (8) in addition to the common reaction of β-hydride transfer to coordinated ethylene, which proceeds during ethylene homopolymerization over TMC by reaction (4) in Scheme 1. Reaction (7) involves the Ti–CH_2_–CH(R)CH_2_P terminal group, which is bound to titanium ion in the active site and forms upon normal 1,2-addition of α-olefin to the growing polymer chain. Reaction (8) involves the Ti–CH(R)–CH_2_P terminal group, which forms after the 2,1-addition of α-olefin to the growing polymer chain. The appearance of these two additional chain transfer reactions results in the formation of copolymers, which have a decreased molecular weight and contain vinylidene and trans-vinylene groups.

**Figure 2:**
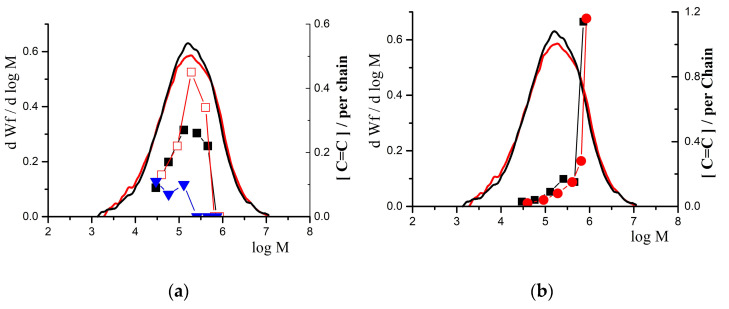
Data on the content of terminal double bonds of different types calculated per one polymer chain for separate fractions of ethylene copolymers with propylene and hexene-1 with close content of branchings (experiments 2 and 5 in Table 5). (**a**) Distribution of terminal vinyl double bonds for ethylene/propylene copolymer (─■─) and ethylene/hexene-1 copolymer (─□─) and distribution of terminal vinylidene double bonds in the ethylene/propylene copolymer (─▼─); (**b**) Distribution of the trans-vinylene double bonds in the ethylene/propylene copolymer (─■─) and ethylene/hexene-1 copolymer (─●─) [24].

In ref. [24], data on the significant heterogeneity of the active centers of TMC in the reaction of the chain transfer with ethylene and additional reactions of the chain transfer with the participation of α-olefins (reactions (7) and (8)) during the copolymerization of ethylene with propylene and hexen-1 were obtained. Copolymers of ethylene and α-olefins were separated into fractions with different molecular weights and narrow MWD, and the content of terminal double bonds of different types in these fractions was determined (Figure 2).

Data presented in Figure 2 reveals an essential heterogeneity in the distribution of vinyl, vinylidene, and trans-vinylene groups for the fractions of copolymers with different molecular weight. Vinyl and vinylidene terminal bonds are present only in the fractions with low and moderate molecular weight (Figure 2a). At the same time, trans-vinylene double bonds are virtually absent in the fractions with low and moderate molecular weight (below 10^5^ g/mol) and are present only in the fractions with a higher molecular weight, predominantly in the fraction with the molecular weight of ca. 7 × 10^5^ g/mol (Figure 2b).

The data presented above testify to the considerable heterogeneity of the TMC active sites in the polymer chain transfer reactions with ethylene (reaction (3)) and polymer chain transfer involving a comonomer, which occurs during ethylene copolymerization with α-olefins (reactions (7) and (8)). The chain transfer with ethylene proceeds predominantly with the participation of the active sites producing the polymer with moderate and low molecular weight. The additional chain transfer reaction with the participation of α-olefin, which proceeds by reaction (7), involves active sites producing the polymer with moderate and low molecular weight. The additional chain transfer with the participation of α-olefin, which proceeds by reaction (8), involves only the active sites producing the polymer with an increased molecular weight. It should be noted that this reaction may occur only after 2,1-addition of α-olefin to the growing polymer chain on the active sites having a low regiospecificity.

### 3.3. Chain Transfer Reaction with Hydrogen in the Polymerization of Ethylene, Propylene, and Hexene-1, and Copolymerization of Ethylene with α-Olefins over Supported Titanium–Magnesium Catalysts

It is well known that hydrogen is the most efficient chain transfer agent at the polymerization of ethylene and α-olefins over supported titanium–magnesium catalysts [8,9,18,69,70,71,72,73,74,75,76,77,78,79,80]. The polymer chain transfer with hydrogen proceeds by reaction (6) in Scheme 1. Titanium hydride formed by this reaction interacts with the monomer, which leads to regeneration of the (Cl_2_Ti–CH_2_R) active site containing the alkyl group.

Our study [29] presents data concerning the effect of hydrogen on the molecular weight and molecular weight distribution of polymers obtained by polymerization of ethylene, propylene, and hexene-1 and copolymerization of ethylene with propylene and hexene-1 over titanium–magnesium catalysts. These data demonstrate that, in some cases, the obtained results are associated with considerable heterogeneity of the TMC active sites in the polymer chain transfer reactions with hydrogen.

#### 3.3.1. The Effect of Hydrogen on the Molecular Weight and MWD of Polymers Produced during Polymerization of Ethylene, Propylene, and Hexene-1

**Figure 3:**
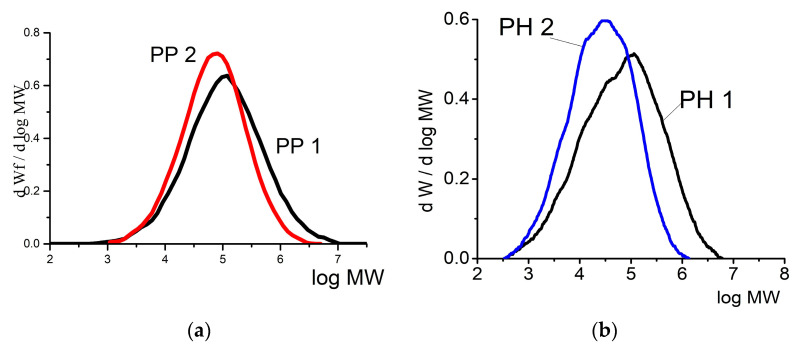
Data MWD curves of the polymers presented in Table 6: (**a**) polypropylene sample PP 1 (polymerization without hydrogen) and PP 2 (polymerization with hydrogen); (**b**) polyhexene sample PH 1 (polymerization without hydrogen) and PH 2 (polymerization with hydrogen) [29].

In the case of ethylene polymerization, the introduction of hydrogen in the gas phase, and the subsequent increase in its content in the reactor up to 50 vol.% leads to the expected decrease in the molecular weight of polyethylene. Therewith, the shape of MWD curves and polydispersity of the produced polymer virtually does not change. These results testify to the homogeneity of the TMC active sites in the chain transfer with hydrogen at ethylene polymerization. However, in the case of propylene and hexene-1 polymerization, we observe the great effect of hydrogen on the polydispersity of polypropylene and polyhexene (Table 6 and Figure 3).

It is seen that propylene and hexene-1 polymerization in the absence of hydrogen leads to the formation of polymers with broad MWD (M_w_/M_n_ = 9.1 for the PP 1 sample and M_w_/M_n_ = 16 for the PH 1 sample, Table 6). The introduction of hydrogen during polymerization results in a significant narrowing of MWD for both polymers (M_w_/M_n_ = 5.2 for the PP 2 sample and M_w_/M_n_ = 6.7 for the PH 2 sample, Table 6). A comparison of MWD curves for the PP 1 and PP 2 samples (Figure 3a) and the PH 1 and PH 2 samples (Figure 3b) shows that the narrowing of MWD occurs mostly due to a decrease in the contribution of the high-molecular-weight components in the PP 1 polymer and the PH 1 polymer.

The results of the MWD curves deconvolution into Flory components for PP 1 and PP 2 samples obtained in the presence or absence of hydrogen are presented in Table 7. It is seen that the introduction of hydrogen into the polymerization leads to a decrease in the number of Flory components from five to four because component V, with a high molecular weight, disappears.

Therefore, the results obtained in the case of propylene and hexene-1 polymerization show the noticeable inhomogeneity of active sites of TMC catalyst in the chain transfer reaction with hydrogen; namely, the active sites that produce polypropylene and polyhexene with the highest molecular weight have the highest reactivity in the chain transfer reaction with hydrogen.

#### 3.3.2. The Effect of Hydrogen on the Molecular Weight and MWD of Copolymers Produced at Ethylene Copolymerization with Propylene and Hexene-1

**Figure 4:**
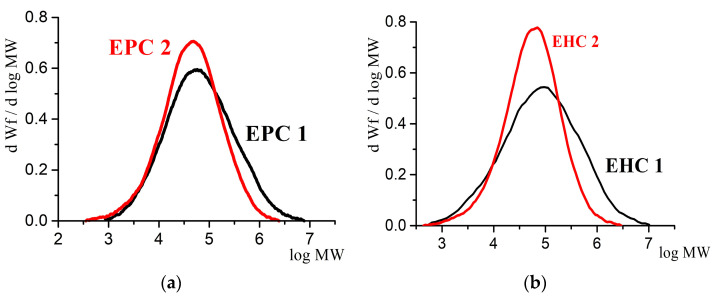
Data MWD curves of copolymers presented in Table 8: (**a**) samples of ethylene/propylene copolymers EPC 1 and EPC 2; (**b**) samples of ethylene/hexene copolymers EHC 1 and EHC 2.

As it was noted in Section 3.3.1, the introduction of hydrogen during the homopolymerization of ethylene leads to a decrease in the molecular weight of the polymer, but it does not affect the polydispersity of the polymer (M_w_/M_n_ values). At the same time, in ref. [29] it was demonstrated that during the copolymerization of ethylene with propylene and hexene-1, the introduction of hydrogen during polymerization leads not only to a decrease in the molecular weight of copolymers, but also to a significant narrowing of MWD (a decrease in M_w_/M_n_ values; Table 8 and Figure 4).

The introduction of a comonomer commonly leads to a pronounced increase in the activity of catalysts in comparison with ethylene homopolymerization [18,32,33,81,82,83]. So, we carried out the copolymerization at a low ethylene pressure (1–2 bar).

It is seen the MWD narrowing occurs due to a decrease in the contribution of the copolymers MWD components with high molecular weight (Figure 4), similar to that observed for the propylene and hexene-1 homopolymerization (Figure 3). This result is clearly supported by the data on deconvolution of MWD curves into Flory components for samples EPC1 and EPC2 (Table 9). One can see that the hydrogen introduction leads to the disappearance of the high molecular weight component (V) in the EPC2 sample.

Thus, it can be concluded that in all cases (homopolymerization of α-olefins and copolymerization of ethylene with α-olefins), the presence of branchings in the growing polymer chain leads to the appearance of heterogeneity of the TMC active sites in the chain transfer reaction with hydrogen. Therewith, in all cases, an increased reactivity toward the chain transfer reaction with hydrogen is observed for the active sites producing the high-molecular-weight component of these polymers, which leads to the narrowing of MWD of the produced polymers.

It was shown in Section 3.2 that a close variant of heterogeneity of active sites was observed for the chain transfer reaction involving α-olefin at ethylene copolymerization with hexene-1 in the absence of hydrogen. In this case, there appears a new route of the chain transfer reaction, which is associated with the appearance of branchings in the growing polymer chain after the 2,1-addition of α-olefin to the growing chain (reaction (8)). This reaction was shown to proceed predominantly on the active sites that produce high-molecular-weight copolymers of ethylene with propylene and hexene-1. As was demonstrated in this Section, in the case of ethylene copolymerization with propylene and hexene-1 in the presence of hydrogen, the chain transfer with hydrogen also proceeds more likely on the TMC active sites producing high-molecular weight polymer (Figure 4).

Earlier, it was found [78,84] that the TMC active sites with a decreased regiospecificity, on which the 2,1-addition of propylene to the growing chain can proceed, possess an increased reactivity toward the chain transfer reaction hydrogen. In this case, it is possible to propose the chain transfer reaction with hydrogen proceeds by two routes (reactions (9) and (10)) with different values of rate constants of the chain transfer reactions with hydrogen (K_tr_^H^ (1) and K_tr_^H^ (2)).

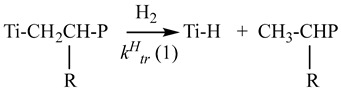
(9)

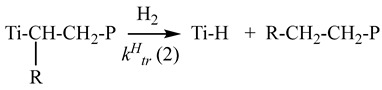
R=CH_3_, C_4_H_9_; P, polymer chain            (10)

Therewith, the K_tr_^H^ (2) value should considerably exceed the K_tr_^H^ (1) value. We believe our results concerning the effect of hydrogen on the molecular weight distribution of polymers with different compositions (polypropylene, polyhexene, and ethylene copolymers with α-olefins), which contain branchings in the growing polymer chain, are consistent with this statement.

## 4. Conclusions

1. The experimental data on the influence of the polymerization conditions of ethylene, propylene, hexene-1, and copolymerization of ethylene with α-olefins on the molecular weight and polydispersity (M_w_/M_n_ values) of polymers obtained on the multisite supported titanium–magnesium catalysts are presented and discussed. The results allow us to identify the heterogeneity of the active centers of these catalysts in chain transfer reactions with an organoaluminum cocatalyst (triethylaluminum), hydrogen, and comonomer in the case of copolymerization of ethylene with α-olefins.

2. In the case of polymerization of ethylene and hexene-1, the chain transfer reaction with TEA takes place with higher reactivity at active centers producing a low-molecular-weight polymer, which leads to an increase in the polydispersity of obtained polymers. A new scheme of two reactions of the limitation of chain propagation with the participation of TEA, which is reversibly adsorbed on the active centers of TMC, is presented.

3. In the case of copolymerization of ethylene with propylene and hexene-1, two additional chain transfer reactions occur with the participation of α-olefins, which lead to a decrease in the molecular weight of the obtained copolymers, compared to homopolyethylene. Schemes of these reactions are proposed based on experimental data on the appearance of terminal vinylidene and trans-vinylene groups in copolymers. It was found that the chain transfer reaction with the formation of terminal vinylidene groups takes place on active centers, producing a copolymer with a low molecular weight (<5 × 10^5^ g/mol), and the chain transfer reaction with the formation of trans-vinylene groups takes place on active centers, producing a high-molecular copolymer (>5 × 10^5^ g/mol).

4. In the case of polymerization of propylene, hexene-1, and copolymerization of ethylene with α-olefins, the chain transfer reaction with hydrogen proceeds with a higher reactivity on active centers that produce high-molecular polymers, which leads to a narrowing of the molecular weight distribution of polymers obtained in the presence of hydrogen. According to the literature data [78,84], these centers have reduced regiospecificity (increased probability of the 2,1-addition of α-olefin to the growing polymer chain), which ensures their increased reactivity in the chain transfer reaction with hydrogen.

## Figures and Tables

**Table 1:** Data on the effect of ethylene pressure and TEA concentration on the molecular weight and polydispersity of polyethylene.

Exp. No.	P C_2_H_4_, bar	(TEA), mmol/L	Polymer Yield ^1^, kgPE/g cat	M_n_, kg/mol	M_w_, kg/mol	M_w_/M_n_
1	1	2.4	0.4	39	430	11.0
2	2	2.4	1.2	76	690	9.1
3	4	2.4	6.1	145	690	4.8
4	4	1.2	6.5	250	910	3.6
5	4	4.8	4.7	93	580	6.2

^1^ Polymerization at 80 °C for 1 h.

**Table 2:** Results of the deconvolution of MWD curves for the PE samples obtained at different ethylene pressures (Table 1) [19].

P (C_2_H_4_), bar		4		1	
Flory Component		Contribution, %	M_w_, kg/mol	Contribution, %	M_w_, kg/mol
I		9.0	3000	6.4	2100
II		28.5	860	23.2	690
III		40.0	330	27.2	275
IV		21.8	100	26.7	100
V		-	-	12.9	30
VI		-	-	3.2	6.8
Sum of components	M_w_, kg/mol	645		410	
	M_w_/M_n_	4.6		9.2	

**Table 3:** Results of the deconvolution of MWD curves for the PE samples obtained at different TEA concentrations (Table 1) [19].

(AlEt_3_), mmol/L		1.2		4.8	
Flory Component		Contribution, %	M_w_, kg/mol	Contribution, %	M_w_, kg/mol
I		8.9	3170	6.6	3500
II		34.6	1100	18.8	1070
III		42.5	470	38.6	390
IV		14.2	135	28.3	120
V				7.7	39
Sum of components	M_w_, kg/mol	905		590	

**Table 4:** Results of the deconvolution of MWD curves for polyhexene samples obtained with TIBA and TEA cocatalysts [66].

		**Cocatalyst**			
		**TIBA**		**TEA**	
M_w_, ^1^ kg/mol		2100		290	
M_w_/M_n_ ^1^		3.1		17	
Flory Component		Contribution, %	M_w_, kg/mol	Contribution, %	M_w_, kg/mol
I		25.4	4700	10	1650
II		60.1	1400	26.4	410
III		14.2	490	34.8	115
IV		-	-	24.7	28
V		-	-	10.4	6
Sum of components	M_w_, kg/mol	2100		300	
	M_w_/M_n_	3.1		16	

^1^ Polymerization at 70 °C, [TEA] = 5 mmol/L, [TIBA] = 6 mmol/L, [C_6_H_12_] = 1 mol/L, for 1 h.

**Table 5:** Data on the molecular weight and polydispersity of copolymers and content of terminal double bonds of different types in copolymers of ethylene with propylene and hexene-1 [24].

Exp. No.	Comonomer	(Cα/C_2_), molar	Polymer Yield ^1^, kg/g cat	M_n_, kg/mol	M_w_, kg/mol	M_w_/M_n_	Cα ^2^, mol. %	Content of Double Bonds ^3^		
								CH_2_=CHR	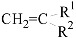	R_1_CH=CHR_2_
1	─	0	1.2	76	690	9.1	─	0.20	0.06	─
2	C_3_H_6_	0.18	4.7	50	420	8.4	2.0	0.14	0.08	0.04
3		0.51	5.5	57	360	6.3	4.9	0.23	0.14	0.10
4	C_6_H_12_	2.3	12.5	59	540	9.2	1.0	0.24	─	0.04
5		4.6	8.7	49	430	8.8	2.3	0.24	─	0.07

^1^ Polymerization at ethylene pressure 2 bar, 80 °C, cocatalyst (AlEt_3_) = 2.6 mmol/L, without hydrogen. ^2^ Comonomer content in copolymers. ^3^ Calculated per one polymer chain.

**Table 6:** Data on the effect of hydrogen on the molecular weight and polydispersity of polypropylene (experiments PP 1 and PP 2) and polyhexene (experiments PH 1 and PH 2) [29].

Exp. No.	H_2_	Polymer Yield, kg/(g_cat_)	M_n_, kg/mol	M_w_, kg/mol	M_w_/M_n_
PP 1 ^1^	-	7.1	34	310	9.1
PP 2 ^1^	+ ^3^	17.8	29	150	5.2
PH 1 ^2^	-	0.5	14	230	16
PH 2 ^2^	+ ^4^	2.1	9.4	63	6.7

^1^ Polymerization at propylene pressure = 6 bar, 70 °C, [TEA] = 4.1 mmol/L, 1 h. ^2^ Polymerization at (C_6_H_12_) = 2 M, 70 °C, (TEA) = 6 mmol/L, for 10 min. ^3^ 2.3 vol.% of H_2_ in gas phase. ^4^ 1 bar of H_2_ in the gas phase.

**Table 7:** Data on the deconvolution of MWD curves into Flory components for the polypropylene samples shown in Figure 3a [29].

Sample ^1^		PP 1		PP 2	
H_2_, vol. %		─		2.3	
Flory Component		Contribution, %	M_w_, kg/mol	Contribution, %	M_w_, kg/mol
I		5.5	7	11.7	19
II		21.6	32	37.1	57
III		40.3	110	37.8	155
IV		29.2	390	13.5	510
V		10.2	1700		
Sum of components	M_w_, kg/mol	320		145	
	M_w_/M_n_	12		4.2	

^1^ Experiment numbers correspond to experiments in Table 6.

**Table 8:** Data on the effect of hydrogen on the molecular weight and polydispersity of copolymers of ethylene with propylene (EPC) and ethylene with hexene-1 (EHC).

Exp. No	C_α_ Content, mol %	P (H_2_), bar	Polymer Yield ^1^, Kg/g_cat_	M_n_, kg/mol	M_w_, kg/mol	M_w_/M_n_
EPC 1 ^1^	25	-	1	23	190	8.3
EPC 2 ^2^	21	0.1	1.4	17	99	5.8
EHC 1 ^3^	2.1	-	3.3	20	280	14.0
EHC 2 ^3^	2.1	0.25	2.0	24	110	4.6

^1^ Polymerization conditions: ethylene pressure 1 bar, propylene pressure 2 bar, 70 °C, for 1 h. ^2^ Polymerization conditions: ethylene pressure 2 bar, propylene pressure 2 bar, 70 °C, for 1 h. ^3^ Polymerization conditions: ethylene pressure = 2 bar, 80 °C, (TEA) = 2.4 mmol/L, (C_6_)/(C_2_)= 2.3 (molar), polymerization duration 15 min.

**Table 9:** Data on the deconvolution of MWD curves into Flory components for ethylene/propylene copolymer samples presented in Figure 4a [29].

Sample ^1^		EPC 1		EPC 2	
P (H_2_),bar		─		0.1	
Flory Component		Contribution, %	M_w_, kg/mol	Contribution, %	M_w_, kg/mol
I		11.6	9	8.3	5.9
II		32.1	32	35.5	26
III		34.2	100	42.3	81
IV		21.5	380	18.3	300
V		6.7	1600		
Sum of components	M_w_,kg/mol	180		95	
	M_w_/M_n_	7.8		5.8	

^1^ Samples numbers correspond to samples in Table 8.

## Data Availability

All needed data are in the Article.
